# Implementing data on targeted therapy from the INFORM registry platform for children with relapsed cancer in Sweden

**DOI:** 10.3389/fonc.2024.1340099

**Published:** 2024-01-31

**Authors:** Sofia Wallin, Ingrid Øra, Gabriela Prochazka, Johanna Sandgren, Caroline Björklund, Gustaf Ljungman, Hartmut Vogt, Torben Ek, Cornelis M. van Tilburg, Anna Nilsson

**Affiliations:** ^1^ Division of Pediatric Oncology, Department of Women and Children´s Health, Karolinska Institutet, Stockholm, Sweden; ^2^ Division of Pediatric Hematology-Oncology, Skåne University Hospital, & Clinical Sciences IKVL, Lund University, Lund, Sweden; ^3^ Department of Oncology-Pathology, Karolinska Institutet, Stockholm, Sweden; ^4^ Clinical Pathology and Cancer Diagnostics, Karolinska University Hospital, Stockholm, Sweden; ^5^ Division of Pediatric Hematology-Oncology, Umeå University Hospital, Umeå, Sweden; ^6^ Department of Women and Children´s Health, Pediatric Hematology-Oncology Uppsala University, Uppsala, Sweden; ^7^ Division of Pediatric Hematology-Oncology B153, Crown Princess Victoria Children’s Hospital, and Division of Children's and Women's Health, Department of Biomedical and Clinical Sciences, Linköping University, Linköping, Sweden; ^8^ University of Gothenburg and Children´s Cancer Center, Sahlgrenska University Hospital, Gothenburg, Sweden; ^9^ Hopp Children’s Cancer Center Heidelberg (KiTZ), Heidelberg, Germany; ^10^ Clinical Cooperation Unit Pediatric Oncology, German Cancer Research Center (DKFZ), Heidelberg, Germany; ^11^ Department of Pediatric Oncology, Hematology, Immunology and Pulmonology, Heidelberg University Hospital, Heidelberg, Germany; ^12^ German Cancer Consortium (DKTK), National Center for Tumor diseases (NCT), Heidelberg, Germany; ^13^ Division of Pediatric Hematology-Oncology, Tema Barn, Astrid Lindgren Children’s Hospital, Stockholm, Sweden

**Keywords:** pediatric oncology, pediatric cancer, precision medicine, molecular diagnostic techniques, molecular targeted therapy

## Abstract

**Background:**

Advances in treatment of childhood malignancies have improved overall cure rates to 80%. Nevertheless, cancer is still the most common cause of childhood mortality in Sweden. The prognosis is particularly poor for relapse of high-risk malignancies. In the international INFORM registry, tumor tissue from patients with relapsed, refractory, or progressive pediatric cancer as well as from very-high risk primary tumors is biologically characterized using next-generation sequencing to identify possible therapeutic targets. We analyzed data from Swedish children included in the INFORM registry concerning patient characteristics, survival, sequencing results and whether targeted treatment was administered to the children based on the molecular findings.

**Methods:**

A registry-based descriptive analysis of 184 patients included in the INFORM registry in Sweden during 2016–2021.

**Results:**

The most common diagnoses were soft tissue and bone sarcomas followed by high grade gliomas [including diffuse intrinsic pontine glioma (DIPG)]. Complete molecular analysis was successful for 203/212 samples originating from 184 patients. In 88% of the samples, at least one actionable target was identified. Highly prioritized targets, according to a preset scale, were identified in 48 (24%) samples from 40 patients and 24 of these patients received matched targeted treatment but only six children within a clinical trial. No statistically significant benefit in terms of overall survival or progression free survival was observed between children treated with matched targeted treatment compared to all others.

**Conclusion:**

This international collaborative study demonstrate feasibility regarding sequencing of pediatric high-risk tumors providing molecular data regarding potential actionable targets to clinicians. For a few individuals the INFORM analysis was of utmost importance and should be regarded as a new standard of care with the potential to guide targeted therapy.

## Introduction

Childhood cancer treatment has continuously improved with significant progress during recent years reaching approximately 80% overall survival. Indeed, survival rates have improved for all pediatric cancers; leukemias, central nervous system (CNS) tumors and other/extra cranial solid tumors and more so for children than for adolescents and adults (1, 2). Despite this achievement using optimized chemotherapy, local treatment with surgery and radiotherapy as well as optimization of supportive care, the prognosis for very high-risk and refractory cancers remains poor. Thus, cancer is still the most common cause of death among children in Sweden (3–5), where patients with relapsed disease encounter survival rates of less than 20%. Moreover, survivors are often facing serious (long term) side effects from current treatments such as cardiac complications, endocrine deficiencies, cognitive problems, and secondary cancers (3). Therefore, to develop more effective and less toxic treatments it is critical to include molecular sequencing to enable treatment with targeted therapy (reviewed in (4), (5)).

Research in the field of molecular biology has led to a significantly increased understanding of cancer biology. Analysis of tumor samples with next-generation sequencing techniques enabling development of new targeted therapies has opened possibilities to tailor individual treatment (6). Cancers in adults are often driven by mutations derived from long time exposure to diverse environmental factors. In contrast, most pediatric cancers carry relatively few background mutations which facilitate identification of tumor driving genetic alterations (7). Although it is yet difficult to draw firm therapeutic conclusions due to limited patient numbers and comparability challenges, this is an area of clinical research that needs to be investigated. Several studies are currently underway worldwide to provide robust data and address the clinical benefit of precision medicine in pediatric oncology (8, 9).

The INFORM program (INdividualized Therapy FOr Relapsed Malignancies in Childhood) was initiated in 2015 in Germany by the German Cancer Research Center (DKFZ) (10) with the aim to characterize tumor material from pediatric patients with treatment-resistant high-risk cancer. In the pilot study, the molecular profiling included whole-exome, low-coverage whole-genome, and RNA sequencing as well as methylation and expression microarray analyses. In parallel, an analytical pathway based on a prioritization algorithm was developed to ensure rapid reporting of potential therapeutic targets for clinical use. Early data from the INFORM program demonstrated the readily available possibilities to perform fast and comprehensive molecular analyses of tumor samples and paired blood samples, further enabling clinical decision-making and the initiation of adequate targeted therapies (10). A separate study also showed how to perform similar analyses on patients with DIPG (Diffuse intrinsic pontine glioma) (11). The INFORM pilot study also enlightened the clinical relevance of re-analysis in the case of tumor recurrence, as some showed significant biological evolution between primary tumor and relapse. It is well documented that genomic characterization of neuroblastomas at the time of diagnosis and at relapse may provide valuable clinical information (12, 13).

Approximately 300 to 350 Swedish children and adolescents (0–18 years of age) are annually diagnosed and treated for cancer at one of the six childhood cancer centers located at regional university hospitals. Approximately 25–30% each are treated in Stockholm and Gothenburg, 15–20% each in Uppsala and Lund and additionally 5–10% each in Linköping and Umeå. The aim of this analysis was to structure and visualize information on the Swedish patient cohort enrolled in the INFORM registry regarding clinical patient data, analysis of target genes and subsequent treatment. Moreover, the study aimed to investigate whether these results were implemented in clinical care of pediatric cancer patients on a national level. A potential clinical benefit of providing targeted treatment was also assessed in this first implementation of precision medicine in Swedish pediatric oncology.

## Methods

### Inclusion criteria for the INFORM registry

The overall patient criteria for inclusion in the INFORM registry study are children and adolescents with treatment-resistant high-risk malignancies, i.e. recurring and/or refractory disease, as described in detail (10). Clinical diagnostic tumor material (retrieved via biopsy) and reference material (blood sample or buccal swab) were obtained; DNA and RNA were prepared at the Swedish Childhood Tumor Biobank (BTB) before shipment to the DKFZ in Heidelberg, Germany for molecular analyses.

The INFORM study for Swedish patients was approved 2016 by the Regional Ethical Review Board in Stockholm (Ref-nr 2016/1782-31) and updated 2022 (Ref-nr 2022-00646-02). The study was conducted in accordance with the declaration of Helsinki. Informed consent from patients and/or legal guardians was obtained before collection of biological material and registration in the study-associated MARVIN database. The study was registered with the German Clinical Trials Register, number DRKS00007623.

The sequencing results and potential targeted treatments were presented and discussed at the weekly INFORM Target decision board during a teleconference with participation of molecular biologists, pediatric oncologists, pharmacologists, the treating physician, and the national PI for the registry in Sweden.

Furthermore, the treating physician had the opportunity to discuss further with other Swedish oncologists or colleagues from the other Nordic countries regarding relevant and matching trials at weekly multidisciplinary videoconferences (NOPHO Match) on demand.

### Patients and data collection

Patient data on sex, age and diagnosis were collected from the MARVIN database. Additional data of interest were time between shipping of tissue samples and presentation of the results at the INFORM target decision board, the origin of the malignant tissue sample, whether complete molecular analysis according to study protocol was performed, quantity and prioritization level (1–7) of identified targets and exact genetic alteration if priority 1–2. Data on whether patients received precision-based treatment and/or other oncological treatments, and survival status was collected from MARVIN.

### Data and statistical analysis

The data/parts of the data from 217 tumor samples evaluated in this study were produced and kindly provided by the INFORM registry (10, 14). Age differences between groups were analyzed using Kruskal-Wallis test followed by multiple comparisons test and data are presented with median (range). Figures and tables were completed using GraphPad Prism.

The overall survival and progression-free survival were illustrated using Kaplan-Meier estimates. Difference between curves were tested using Log-Rank test. The analysis of progression-free survival was repeated using cumulative incidence and treating death as a competing risk to progression. Difference in cause-specific cumulative incidence function were analyzed using Gray’s test (15) and this was done using “R” (16).

## Results

### Patient characteristics and national coverage in the INFORM registry

**Figure 1:**
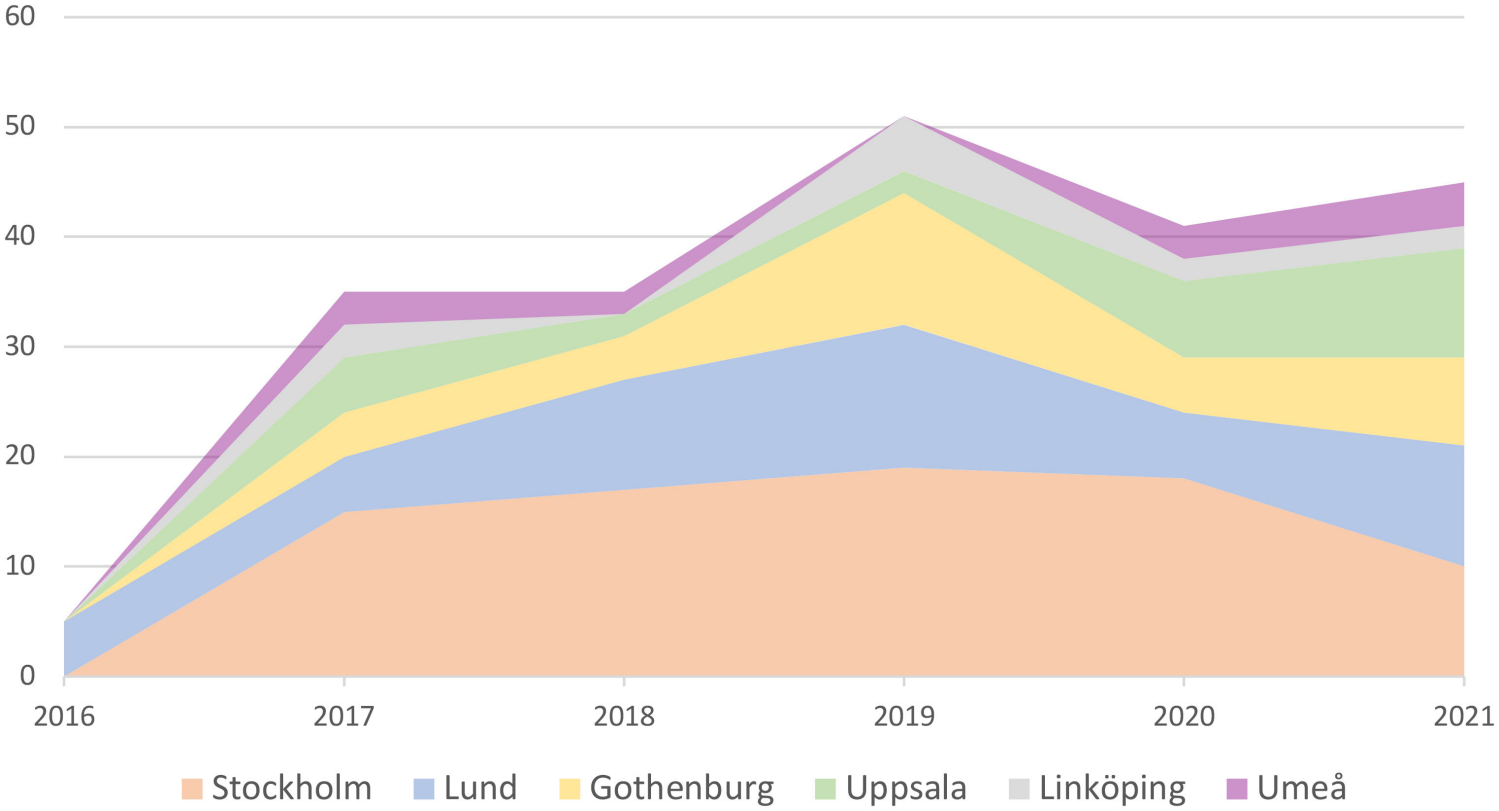
Inclusion rate in the INFORM registry from the six Swedish pediatric cancer centers 2016–2021, 18 cases were included at more than 1 relapse.

Patient tumor samples (n=217) were included in the INFORM registry during 2016-2021 from six university hospitals treating children with cancer in Sweden: Karolinska University Hospital, Stockholm (n=79 samples), Uppsala University Children´s Hospital (n=29), Lund University Hospital (n=51), Queen Silvia Children’s Hospital, Gothenburg (n=34), Crown Princess Victoria Children’s Hospital, Linköping (n=12) and Norrland University Hospital, Umeå (n=12) (**Figure 1**). Five cases were not analyzed: four due to insufficient amount of tumor DNA/RNA and one for not meeting the inclusion criteria for the study. Twenty-five patients were registered at subsequent relapse(s) and thus, the study cohort involves 184 unique subjects. The reason for inclusion was due to cancer relapse in 147 children, refractory disease in 20 children and 17 children were included at primary diagnosis.

Patient characteristics are displayed in **Table 1**. The distribution of pediatric malignancies in the cohort was 62% solid tumors, 30% CNS tumors, 5% hematological malignancies and 3% others (**Figures 2A, B**). The small group “others” (3%) included children with infantile myofibromatosis, Langerhans cell histiocytosis and juvenile ossifying fibroma. The median age of the entire cohort was 13 years although 20 patients were older than 18 years, of which the primary diagnosis of the cancer had occurred at < 18 years of age. Children with solid tumors were significantly older than children in the group “others” [14 years (9-26) vs. 4 years (0-8), P=0.04]. The proportion of female patients was slightly higher than the proportion of male patients (53 vs. 47%), without any significant differences between groups. Twenty-two subjects were included twice, and three subjects were included three times. Most patients were included at relapse (80%) and the number of individual relapses varied between one and eight. The general condition of patients was assessed using the Karnofsky/Lansky score at inclusion and the median value was 90% for the whole cohort and the respective disease groups. This score decreased from the first inclusion in INFORM to the subsequent registrations at later relapse to a median of 80% (P>0.5).

**Table 1:** Patient characteristics of 184 Swedish patients included in the INFORM registry 2016–2021.

Diagnosis (n)	
Solid tumor	115
CNS tumor	55
Leukemia/Lymphoma	9
Other	5
Age in years (median, range)	
All patients	13 (0–26)
Solid tumor	14 (0–26)
CNS tumor	11 (2–21)
Leukemia/Lymphoma	9 (1–17)
Other	1 (0–8)
Female/male (n)	
All patients	97/87
Solid tumor	62/53
CNS tumor	26/29
Leukemia/Lymphoma	5/4
Other	4/1
Disease status	
Relapse	147
Primary disease	17
Refractory disease	20
Karnofsky/Lansky score (n)#	
50%	11
60%	8
70%	19
80%	40
90%	70
100%	36
Localization of tissue sample*	
Primary tumor	116
Metastasis	96

* In total 212 samples from 184 unique patients.

# Inclusion criteria Karnofsky/Lansky ≥50.

**Figure 2:**
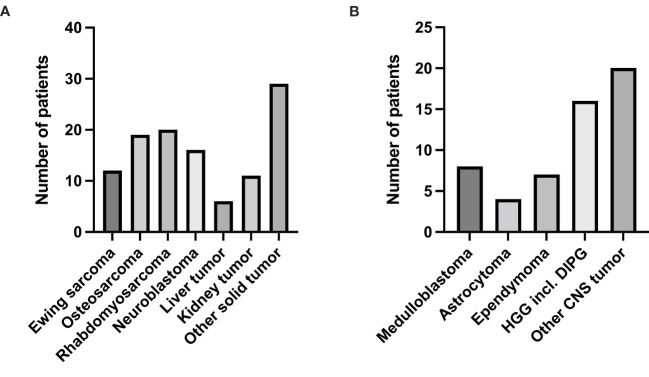
Diagnosis distribution in the two largest disease groups. **(A)** Number of patients with solid tumors. **(B)** Number of patients with CNS tumors.

### Sequencing results and distribution of targets

**Figure 3:**
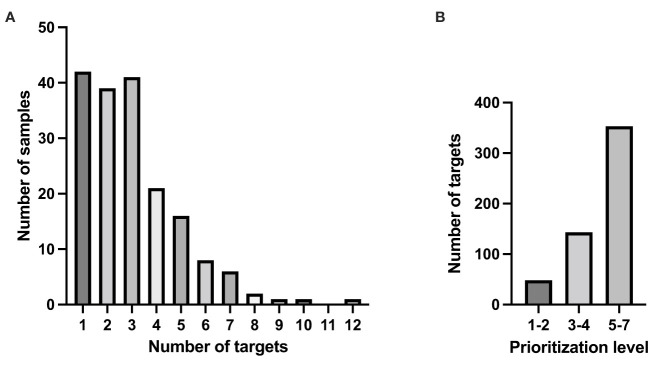
Number of defined targets for treatment in the Swedish study cohort. **(A)** Number of targets among the samples, median=4. **(B)** Number of targets with a prioritization level 1–2, 3–4 and 5–7.

The median turnover time, from shipment of tumor and reference DNA/RNA material to presentation at the INFORM target board was 28 days (range 16-105 days) for samples in the Swedish cohort. Complete molecular genetic analyses were performed on 203 out of 212 patient samples. Nine samples were not analyzed: six deaths occurred before sample analysis. Identified targets were classified according to a seven-grade scale prioritization algorithm by the INFORM registry interdisciplinary review board as previously described (10). At least one actionable target was found in 178 out of the 203 analyzed samples (88%) (corresponding to 157 unique patients) and the median value was 4 targets (range 0-12) per patient (**Figure 3A**). At least one actionable target with a priority score of 1-4 was reported for 110 individuals (60%) and a priority target score 1-2 (corresponding to very high and high) was reported in 48 samples (24%) corresponding to 40 unique patients (**Figure 3B**). The molecular alterations with a target score 1-2 are presented in **Table 2**. The most frequent level 1 gene targets (n=20) were *NTRK* (n=4), *BRAF* (n=6) and *ALK* (n=3). Targets with prioritization level 2 (n=28) were found in *NRAS* and *KRAS* (n=10), *CDK4* (n=4) but also *NTRK* (n=2) and *NF1* (n=5). It was also noted that the molecular profile of tumors could change between the first and subsequent analyses in INFORM (**Supplementary Table 1**).

**Table 2:** Targets with priority level 1-2.

Alteration	Alteration type	Detection method	Prioritiy level	Sex (M/F), diagnosis	Targeted drug therapy
*ALK*	SNV	WES	1	F, Neuroblastoma	Lorlatinib
*ALK*	SNV	WES	1	F, Neuroblastoma	Lorlatinib
*ALK*	SNV	WES	1	M, Neuroblastoma	Crizotinib, Lorlatinib
*BRAF*	SNV	WES	1	F, Tectal glioma	–
*BRAF*	SNV	WES	1	F, HGG	Dabrafenib, Trametinib
*BRAF*	SNV	WES	1	F, Langerhans cell histiocytosis	–
*BRAF*	SNV	WES	1	M, Anaplastic astrocytoma	Dabrafenib, Trametinib
*BRAF*	SNV	WES	1	F, HGG	Everolimus, Trametinib
*CAPZA2:MET*	fusion	RNA-seq	1	M, DIPG	Capmatinib
*ETV6:NTRK3*	fusion	RNA-seq	1	F, Papillary thyroid cancer	Larotrectinib
*ETV6:NTRK3*	fusion	RNA-seq	1	F, Inflammatory myofibroblastic tumor	–
*FGFR1*	other	LC-WGS	1	F, Astrocytic neoplasm	–
*FGFR1*	SNV	WES	1	F, Pilomyxoid astrocytoma	–
*KIAA1549:BRAF*	fusion	RNA-seq	1	M, Optic nerve glioma	–
*KRAS*	SNV	WES	1	F, ERMS	Trametinib
*LMNA:NTRK1*	fusion	RNA-seq	1	M, Spindle cell sarcoma	Larotrectinib
*NRAS*	SNV	WES	1	M, ERMS	Trametinib
*PDGFRA*	SNV	WES	1	M, Anaplastic astrocytoma	–
*QKI:NTRK2*	fusion	RNA-seq	1	F, Low grade glioneural tumor	Larotrectinib
*TGF:ROS1*	fusion	RNA-seq	1	F, Congenital infantile myofibromatosis	Crizotinib
*CDK4*	amplification	LC-WGS	2	F, ARMS	-
*CDK4*	amplification	LC-WGS	2	F, Neuroblastoma	-
*CDK4*	amplification	LC-WGS	2	F, Rhabdomyosarcoma	-
*CDK4*	amplification	LC-WGS	2	M, ARMS	–
*CDK6*	other	LC-WGS	2	F, Medulloblastoma	Ribociclib
*CDKN2A/B*	deletion	LC-WGS	2	M, Anaplastic astrocytoma	Dabrafenib, Trametinib
*DLG1:PIK3CA*	fusion	RNA-seq	2	M, HGG	Trametinib
*FGFR1*	SNV	WES	2	M, Neuroblastoma	–
*FGFR4*	SNV	WES	2	M, ARMS	–
*KRAS*	amplification	LC-WGS	2	M, Mixed germ cell tumor	–
*KRAS*	SNV	WES	2	F, ERMS	–
*KRAS*	SNV	WES	2	M, Peripheral T-cell lymphoma	Trametinib, Valproic acid
*KRAS*	SNV	WES	2	F, ALL	Inotuzumab
*KRAS*	SNV	WES	2	F, ALL	–
*LMNA:NTRK1*	fusion	RNA-seq	2	M, Spindle cell sarcoma	Larotrectinib
*NF1*	InDel	WES	2	M, HGG	Trametinib
*NF1*	other	LC-WGS	2	F, MPNST	Trametinib
*NF1*	InDel	WES/LC-WGS	2	F, HGG	Everolimus
*NF1*	SNV	WES	2	M, ALL	Trametinib
*NF1*	InDel	WES	2	M, ALL	Trametinib
*NRAS*	SNV	WES	2	M, HGG	Trametinib
*NRAS*	SNV	WES	2	M, ARMS	–
*NRAS*	SNV	WES	2	F, ERMS	–
*NRAS*	SNV	WES	2	F, ERMS	Trametinib
*NRAS*	SNV	WES	2	M, Spindle cell sarcoma	Trametinib
*PIK3CA*	SNV	WES	2	M, Neuroblastoma	–
*PIK3CA*	SNV	WES	2	M, Neuroblastoma	Rapamune
*TPR:NTRK1*	fusion	RNA-seq	2	F, MPNST	Larotrectinib

ALL, Acute lymphocytic leukemia; ARMS, Alveolar rhabdomyosarcoma; DIPG, Diffuse intrinsic pontine glioma; ERMS, Embryonal rhabdomyosarcoma; HGG, High-grade glioma; LC-WGS, Low coverage - whole genome sequencing; MPNST, Malignant peripheral nerve sheet tumor; RNA-seq, Ribonucleic acid sequencing; SNV, Single nucleotide variant; WES, Whole exome sequencing.

### Targeted treatment in the whole cohort

In total 70/184 patients (38%) were prescribed targeted treatment and 13 were included in a clinical phase I/II study based on the data generated from the INFORM registry. Among the 40 individuals with a highly prioritized target (level 1-2), 24 patients (60%) were prescribed targeted treatment based on the INFORM data. Out of the 24 patients, only six patients received targeted treatment (larotrectinib, dabrafenib and trametinib) within a clinical phase I/II study based on the molecular profile of the tumor. The remaining 18 patients had matched targeted drug treatment via compassionate use programs or off-label, consisting of trametinib (n=11), larotrectinib (n=5), crizotinib (n=2), everolimus (n=2), dabrafenib (n=2), ribociclib (n=1), lorlatinib (n=1), capmatinib (n=1), rapamune (n=1) and inotuzumab (n=1).

The remaining patients included in INFORM registry received conventional treatment such as drug therapies, radiotherapy, or surgery. In most cases, those receiving targeted therapies had one or more of the conventional cancer drugs at some time-point during follow up due to progressive disease. Twelve patients went to symptomatic palliative care without any further treatment for the relapse.

### Treatment and outcome for relapsed patients

**Figure 4:**
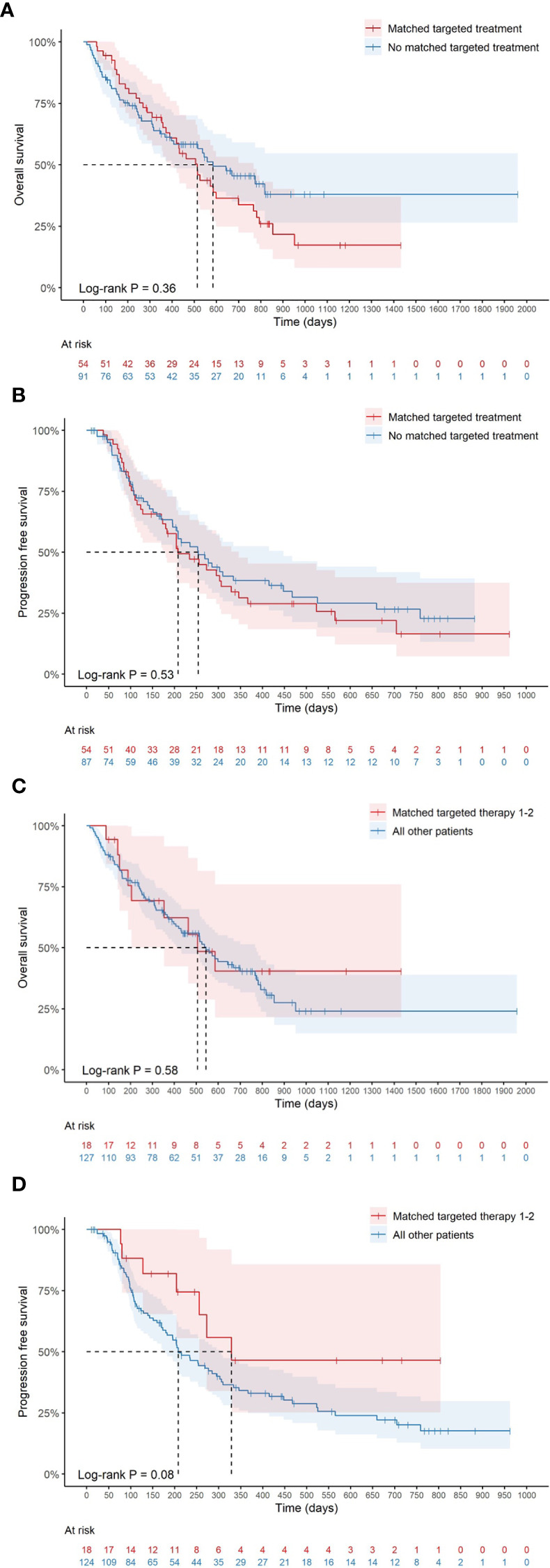
Survival analyses (Kaplan-Meier) where median time of survival for duplicates/triplicates was measured from the last registration. **(A)** Median overall survival (OS) for patients receiving matched therapy compared to all other patients and in **(B)** median progression free survival (PFS) is shown. In **(C)** median OS overall survival is shown for those children with matched targeted therapy 1-2 and in **(D)** median PFS is displayed for the same group.

The active follow-up period in the INFORM registry study (14) is set to approximately 2 years although patients living longer were followed longer. As the INFORM registry primarily focuses on patients at relapse, the outcome for the Swedish cohort was evaluated only for children included at relapse (n=147 with data missing for 2 individuals) from the time-point of first registration in MARVIN to the event of interest. Initially, relapsed patients who were provided matched targeted therapy (MTT) (n=54) were compared to those relapsed patients (n=91) who did not receive targeted treatment (**Figures 4A, B**). Median overall survival (OS) in the MTT group was 514 days (95% CI, 389- 767) compared to 584 days [95% confidence interval (CI), 407-not applicable (NA)] in the latter group. No difference in median progression free survival (PFS) was observed between the groups [208 days (95% CI, 173-346) and 254 days (95% CI, 196-448) respectively].

Furthermore, patients with priority targets 1-2 (MTT 1-2; n=18) were compared to remaining patients (n=127) (**Figures 4C, D**). No significant difference in OS was observed for the MTT 1-2 group compared to remaining patients [506 days (95% CI, 353-NA) and 544 days (95% CI, 420-767) respectively, log rank test p=0.58]. In the MTT 1-2 group, there were three children harboring NTRK fusions and two with BRAFV600E mutations who are long term survivors after targeted therapy for more than 2 years. Patients in the MTT 1-2 group had a trend to longer PFS than the remaining patients [329 days, 95% CI, 256-NA) and 208 days (95% CI, 180-303) respectively, log rank p=0.08].

As death may occur before progression of a malignant disease in each patient, these events were considered as competing events when analyzing PFS. The cumulative incidence of the specific events of interest may be informative since ignoring competing events may lead to informative censoring and therefore incorrect estimates of the main event of interest. In **Table 3**, the cumulative incidence for death and progression, respectively, is shown for the MTT 1-2 group and all other patients. While no difference was observed in the 1-year cumulative incidence of death (p=0.4), there was a trend towards decreased 1-year cumulative incidence of progression in the MTT 1-2 group (p=0.068).

**Table 3:** Cumulative incidence of death or progression in patients with level 1-2 targets receiving therapy compared to all other patients.

Outcome	1-year cum inc (95% CI)	P-value
Death		
*Others* *MTT 1-2*	*9,5% (5.0%, 16%)* *18% (4.0%, 39%)*	0.4
Progression		
*Others* *MTT 1-2*	*61% (51%, 69%)* *46% (19%, 69%)*	0.068

## Discussion

Pediatric cancer patients at relapse have limited effective treatment options. The INFORM registry uses next-generation sequencing, to detect actionable molecular alterations as a step towards precision medicine (10). The current study analyzed the Swedish patient cohort included in the INFORM registry regarding clinical data, molecular analysis of tumor samples, the subsequent treatment and survival. Our data demonstrate that sample preparation, shipment and reporting was feasible and timely in this international setting and that the molecular analyses also provided important clinical information. The Swedish cohort shares similarities with the international INFORM registry cohort presented earlier (10, 14). Sweden and Germany have much in common and are both partners in the EU, but still health systems, health financing, regulations governing health and much more differ. In terms of survival on a group basis, no clinical benefit of targeted treatment could be observed although patients with high priority targets treated with a matched drug had a trend towards a longer PFS. Individual patients have achieved long term benefits from molecular screening followed by targeted therapy, especially when harboring druggable targets as NTRK fusions and BRAFV600E mutations.

The Swedish INFORM cohort as well as the previously reported INFORM registry cohort included mainly CNS tumors and sarcomas (14) whereas only few leukemia cases were included despite being the most common childhood cancer diagnosis. The average age at pediatric cancer diagnosis is during preschool, yet the patients included in the INFORM registry were older as most of them had undergone 1^st^ and 2^nd^ line of treatment before inclusion. In addition, several treatment-resistant cancers, such as Ewing sarcoma and osteosarcoma, occur in the adolescence period. Most patients were in good general condition (assessed by the Karnofsky/Lansky score) at study inclusion despite that most children were included at relapse. Patient inclusion in the INFORM registry was not evenly spread between the pediatric oncology centers in Sweden and did not reflect the number of cases the center cares for. In the first years of the study, the centers with the closest collaboration with the national coordinating center enrolled the largest number of patients.

Pharmacologically actionable targets were detected in 88% of the patient samples in the Swedish cohort. A potentially druggable target with a priority level of 1–4 was present in approximately 60% of patients as compared to 67% in the latest publication from the INFORM registry (14). Similar numbers in terms of priority targets have also been reported from the European Mappyacts trial (17) although lower compared to the molecular profiling platform ZERO that presented with 71.4% (18). In the current cohort, actionable targets were not present at the first analysis but detected at later timepoints which is in accordance with previously published papers (9 (17). We demonstrate that the INFORM registry may impact treatment and that renewed biopsies in patients with chemo-resistant relapse is mandatory for potential personalized targeting treatment. In tumors with priority level 1 targets, several known actionable targets such as *ALK*, *BRAF* and *NTRK* were detected in extracranial solid and CNS tumors. In addition, targetable mutations as well as fusion genes need to be analyzed as they provide therapeutic options for pediatric cancer (19), such as *BRAF* fusions/mutation in low-grade gliomas and *NTRK* fusions in several tissue types showing promising results in pediatric clinical trials (20).

Two thirds of patients with high priority targets (1, 2) were treated with targeted drugs either included in a phase I/II trial or provided by pharma. This may reflect a lack of early phase clinical studies for children with cancer in Sweden or reluctancy to refer very sick children to other national study centers or abroad for participation in phase I/II trials. Possibly, some patients with advanced disease may not have been eligible to trials or to novel treatments due to a poor general condition although most patients were in good general condition (assessed by the Karnofsky/Lansky score) at study inclusion. In total, only 13 out of 70 patients who received targeted treatments were enrolled in a clinical trial. The remaining 57 patients where no trials were available received targeted therapies prescribed off label, or by compassionate use programs.

The patients included in the INFORM registry suffer from treatment resistant diseases at a stage where the chance of curative treatment is very low. Relapse of soft tissue and bone sarcomas is associated with a particularly poor prognosis, and despite targeted treatment no long-time survivors were observed. In the most recent article summarizing more than 500 patients in the INFORM registry, an increased PFS for patients with targets prioritized to the highest level 1 who received targeted treatment was shown when compared to all other patients (14). In the same cohort, no significant difference was observed in OS. In the presented Swedish cohort, no significant difference in OS was observed between patients with priority targets who received targeted therapy (MTT 1-2) compared to all other patients. However, there was a trend for a longer PFS in this group of children compared to all other patients. This finding was supported by the lower cumulative 1-year incidence of progression in the MTT 1-2 group. Contrary to the previously published paper from the INFORM registry (14), patients who had tumors with both actionable target score 1-2 were included in our survival analyses.

It is important to keep in mind that the INFORM registry determines potentially treatable targets, but as reported for many other cancers, single agents do not, with only rare exceptions induce sustainable response or cure. Many cancer phenotypes, including those influencing response to therapy are in addition determined by non-genetic mechanisms in addition to genetic alterations such mutations/translocations concerning *BRAF*, *ALK* and *NTRK* (20).

This study describing data on Swedish patients included in the INFORM registry has several limitations. Foremost, the patients were included in a non-interventional registry where any decision on treatment was left to the treating physician at the different sites. The limited numbers of patients and the small fraction of children with priority targets 1-2 hampers the statistical analyses on PFS and OS. We cannot exclude that some children who received a matched targeted drug also received other concomitant treatment. In addition, data management was done at the different pediatric cancer centers and data quality, consistency and accuracy in reporting may have differed. On the other hand, a strength in this study is the low number of children who were lost to follow up.

This study, as well as previous studies, has demonstrated the feasibility of sequencing pediatric tumor material and to provide data on molecular findings and potentially actionable targets to clinicians. However, as opposed to adult oncology, there is a lack of innovative trials, specific biomarker research, immunotherapy, and drugs targeting pediatric cancers. Consequently, a low number of patients in Sweden were enrolled in a clinical trial with monotherapy at relapse which also was shown for the INFORM registry in total (14). As targeted monotherapy in late-stage disease seldom provides a durable response (4, 5, 14, 17), it is not surprising that there was no survival benefit for the children in our cohort.

However, molecular profiling at diagnosis is now offered to newly diagnosed patients in Sweden within the frame of the precision medicine program Genomic Medicine Sweden (21, 22). This may provide an opportunity to apply targeted therapy already at primary diagnosis and in combination with other treatment modalities. Pediatric precision oncology has moved slowly but is now gaining momentum world-wide with different technical and analytic approaches. To further gain impact on patient survival will require a combination of continuous preclinical testing in relevant pediatric tumor models as well as biomarker driven multinational clinical trials. In addition, the different national molecular profiling platforms should team up to provide a robust and sustainable framework, covering all components from technology development to the design and execution of clinical trials as outlined by the Swedish and German centers for personalized medicine (23, 24).

## Data availability statement

The datasets presented in this study can be found in online repositories. The names of the repository and accession number can be found below: European Genome Archive, accession number EGAS00001005112.

## Ethics statement

The studies involving humans were approved by Swedish Ethical Review Authority, Uppsala, Sweden. The studies were conducted in accordance with the local legislation and institutional requirements. Written informed consent for participation in this study was provided by the participants' legal guardians/next of kin.

## Author contributions

SW: Data curation, Formal analysis, Investigation, Writing – original draft. IØ: Data curation, Investigation, Project administration, Writing – review & editing. GP: Investigation, Methodology, Writing – review & editing. JS: Methodology, Resources, Writing – review & editing. CB: Investigation, Writing – review & editing. GL: Investigation, Writing – review & editing, Validation. HV: Investigation, Writing – review & editing. TE: Investigation, Writing – review & editing. CV: Conceptualization, Formal analysis, Funding acquisition, Investigation, Methodology, Project administration, Resources, Writing – review & editing. AN: Conceptualization, Data curation, Formal analysis, Funding acquisition, Investigation, Project administration, Writing – review & editing.
