# Supplementary material for: Implementing data on targeted therapy from the INFORM registry platform for children with relapsed cancer in Sweden
Source: Front Oncol. 2024 Jan 31;14:1340099. doi: 10.3389/fonc.2024.1340099 (PMC10865092; doi:10.3389/fonc.2024.1340099)
Supplement: Supplementary file 1 [file Table_1.docx]

**SUPPLEMENTARY MATERIAL**

**SUPPLEMENTAL TABLE 1.** Targets with priority level 1-4 in patients with two or three analyses

ALL, Acute lymphocytic leukemia; ARMS, Alveolar rhabdomyosarcoma; DSRCT, Desmoplastic small round cell tumor; ERMS, Embryonal rhabdomyosarcoma; HGG, High-grade glioma; MPNST, Malignant peripheral nerve sheet tumor; ND, not done.

|  | **First analysis** | | | **Second analysis** | | | **Third analysis** | | |
| --- | --- | --- | --- | --- | --- | --- | --- | --- | --- |
| **Sex (M/F), diagnosis** | **Gene** | **Priority level** | **Targeted drug therapy** | **Gene** | **Priority level** | **Targeted drug therapy** | **Gene** | **Priority level** | **Targeted drug therapy** |
| M, DSRCT | *CDKN2A/B* | 4 | Pazopanib, Ribociclib | *CDKN2A/B* | 4 | Ribociclib | ND | - | - |
| F, ERMS | *KRAS* | 1 | Trametinib | *KRAS* | 2 | - | ND | - | - |
| M, Synovial sarcoma | *SS18:SSXB2B* | 4 | - | *SS18:SSXB2B* | 4 | - | ND | - | - |
| M, Anaplastic ependymoma | *-* | - | - | *CDKN2A/B* | 3 | - | ND | - | - |
| M, Neuroblastoma | *MYCN* | 4 | Valproic acid | *PIK3CA* | 2 | - | *PIK3CA* | 2 | Rapamune |
|  |  |  |  | *MYCN* | 4 |  | *MYCN* | 4 |  |
| F, Ewing sarcoma | *CDKN2A/B* | 3 | - | *CDKN2A/B* | 3 | - | ND | - | - |
| F, ERMS | *-* | - | Dasatinib | *IGF2* | 3 | Dasatinib | ND | - | - |
| F, MPNST | *TPR:NTRK1* | 2 | Larotrectinib | *PIK3CA* | 4 | Larotrectinib | ND | - | - |
| M, ERMS | *-* | - | - | *NF1* | 3 | - | ND | - | - |
| M, Anaplastic ependymoma | *CDKN2A/B* | 3 | - | *CDKN2A/B* | 3 | Dasatinib, Everolimus, Ribociclib | ND | - | - |
| F, HGG | *BRAF* | 3 | Dabrafenib, Trametinib | *BRAF* | 1 | Dabrafenib, Trametinib | *BRAF* | 1 | Everolimus, Trametinib |
|  |  |  |  | *AXL* | 4 |  | *TSC2* | 4 |  |
| M, Osteosarcoma | *PTEN* | 3 | - | *PTEN* | 3 | Regorafenib | ND | - | - |
| M, Osteosarcoma | *MYC* | 4 | - | *CDKN2A/B* | 3 | - | ND | - | - |
|  | *CCND2* | 4 |  |  |  |  |  |  |  |
| F, Spindle cell sarcoma | *-* | - | - | *CDKN2A/B* | 3 | - | ND | - | - |
|  |  |  |  | *RAF1* | 4 |  |  |  |  |
| F, Osteosarcoma | *MYC* | 4 | Regorafenib | *MYC* | 4 | Nivolumab | ND | - | - |
|  |  |  |  | Immunogenicity | 4 |  |  |  |  |
| F, Rhabdoid meningioma | *FLT1* | 4 | - | *CDKN2A/B* | 3 | Palbociclib, Nivolumab | ND | - | - |
|  | Immunogenicity | 4 |  | *FLT1* | 4 |  |  |  |  |
|  |  |  |  | Immunogenicity | 4 |  |  |  |  |
| M, Ependymoma | *CDKN2A/B* | 3 | - | *CDKN2A/B* | 3 | Ribociclib | ND | - | - |
| M, Peripheral T-cell lymphoma | *KRAS* | 2 | Trametinib, Valproic acid | *-* | - | - | ND | - | - |
| F, ERMS | *NRAS* | 2 | - | *NRAS* | 2 | Trametinib | ND | - | - |
|  | *CDKN2A/B* | 3 |  | *CDKN2A/B* | 3 |  |  |  |  |
| F, ALL | *KRAS* | 2 | - | *KRAS* | 2 | Inotuzumab, Car-T | ND | - | - |
|  | *CDKN2A/B* | 3 |  | *CDKN2A/B* | 3 |  |  |  |  |
|  | Immunogenicity | 4 |  | *FLT3* | 3 |  |  |  |  |
|  | *MAP2K1* | 4 |  | Immunogenicity | 4 |  |  |  |  |
|  |  |  |  | *NF1* | 4 |  |  |  |  |
